# Retracted Article: Physics of excitons and their transport in two dimensional transition metal dichalcogenide semiconductors

**DOI:** 10.1039/c9ra03769a

**Published:** 2019-08-16

**Authors:** Bhaskar Kaviraj, Dhirendra Sahoo

**Affiliations:** Department of Physics, School of Natural Sciences, Shiv Nadar University NH91, Gautam Budh Nagar Greater Noida Uttar Pradesh 201314 India bhaskar.kaviraj@snu.edu.in

## Abstract

Two-dimensional (2D) group-VI transition metal dichalcogenide (TMD) semiconductors, such as MoS_2_, MoSe_2_, WS_2_ and others manifest strong light matter coupling and exhibit direct band gaps which lie in the visible and infrared spectral regimes. These properties make them potentially interesting candidates for applications in optics and optoelectronics. The excitons found in these materials are tightly bound and dominate the optical response, even at room temperatures. Large binding energies and unique exciton fine structure make these materials an ideal platform to study exciton behaviors in two-dimensional systems. This review article mainly focuses on studies of mechanisms that control dynamics of excitons in 2D systems – an area where there remains a lack of consensus in spite of extensive research. Firstly, we focus on the kinetics of dark and bright excitons based on a rate equation model and discuss on the role of previous ‘unsuspected’ dark excitons in controlling valley polarization. Intrinsically, dark and bright exciton energy splitting plays a key role in modulating the dynamics. In the second part, we review the excitation energy-dependent possible characteristic relaxation pathways of photoexcited carriers in monolayer and bilayer systems. In the third part, we review the extrinsic factors, in particular the defects that are so prevalent in single layer TMDs, affecting exciton dynamics, transport and non-radiative recombination such as exciton–exciton annihilation. Lastly, the optical response due to pump-induced changes in TMD monolayers have been reviewed using femtosecond pump–probe spectroscopy which facilitates the analysis of underlying physical process *just* after the excitation.

## Abstract 2

Two-dimensional (2D) group-VI transition metal dichalcogenide (TMD) semiconductors, such as MoS_2_, MoSe_2_, WS_2_ and others manifest strong light matter coupling and exhibit direct band gaps which lie in the visible and infrared spectral regimes.

## Graphical abstract

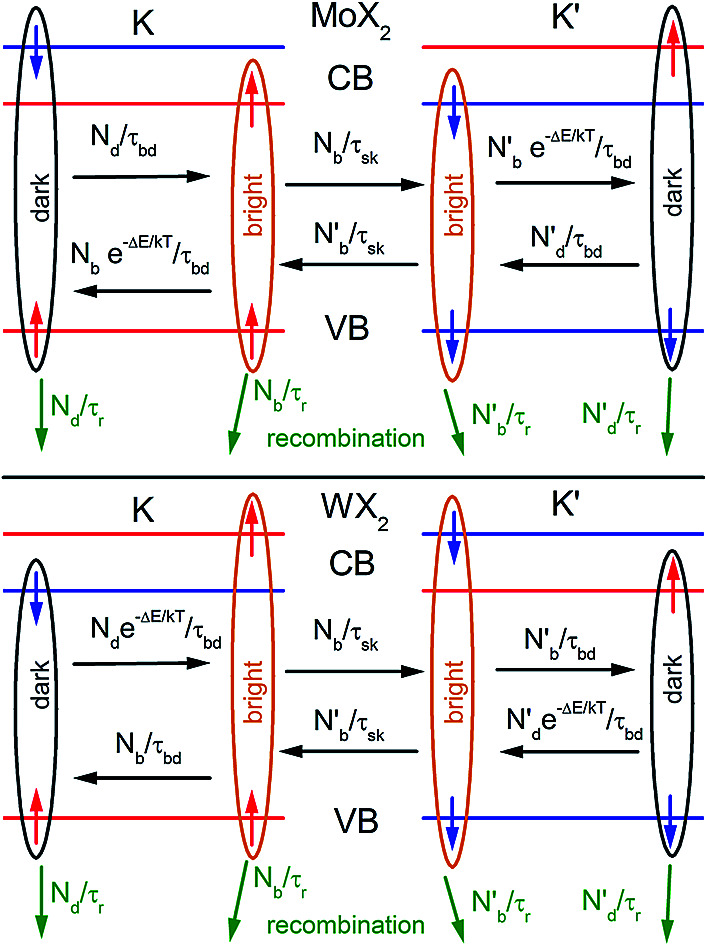

## 1. Introduction

Atomically thin transition metal dichalcogenides (TMDs) are one of the important subclasses of two-dimensional (2D) materials with a wide range of physical properties and potential applications. Their general chemical formula is MX_2_, where M is a transition metal and X a chalcogen atom. Within this exhaustive family of TMDs, some materials have been identified as semiconductors with band gaps in the infrared and visible spectral regions with strong light–matter coupling, making them very useful for applications in optics and optoelectronics. The properties of 2D layers with atomic scale thicknesses differ dramatically from their bulk counterpart,^1,2^ which is one of the primary reasons for renewed interest in this class of materials.

In this article, we review the intrinsic and extrinsic factors that control exciton dynamics and transport in 2D semiconducting TMDs. We restrict ourselves to group-VI TMDs (M = Mo, W; X = S, Se, Te) in the semiconducting 2H phase, as these are the most studied and understood. The 2D character and weak dielectric screening is responsible for enhanced Coulomb interactions in these materials, resulting in the formation of strongly bound electron–hole pairs (excitons) upon optical excitation. In atomically thin layers, particularly monolayers, much progress has been made in the past few years in understanding of exciton physics. In 2005, after the discovery of graphene by Novoselov and Geim,^3^ they showed that the same technique can be applied to isolate other monolayers, in particular a 2D semiconductor – molybdenum disulfide (MoS_2_).^4^ In 2010, Heinz and Wang discovered experimentally and independently that MoS_2_ monolayer exhibits a direct band gap and strong photoluminescence emission.^5,6^ Although, earlier, it was recognized that the optical response of TMDs arises from excitons, it took few more years to get the confirmation from quantitative measurements of the binding energy.^7–9^ Other exciton complexes that were studied, in parallel, were trions (charged excitons)^10–12^ and biexcitons.^13,14^ The study of valley selective optical excitation^15–20^ gave rise to TMD-based valleytronics tracing back to 2012. The advent of van der Waals (vdW) heterostructures, since 2013, permitted the fabrication of TMD heterostructures and the observation of interlayer excitons.^20,21^ The role of dark exciton states has also become an active field of research recently^22–24^ as well as the emission of quantum light from localized states.^25–29^

The research on the fundamental optical properties of TMDs led to the realization of first MoS_2_ monolayer based field effect transistor, demonstrated in 2011 by Kis and colleagues.^30^ The same device structure, a year later, had been used to develop atomically thin phototransistors.^31,32^ Fundamental studies on photoconductivities on TMD bulk^1^ and monolayers^2^ date back earlier. Atomically thin p–n junctions were first realized in 2014, both in lateral^33–35^ and vertical^36–38^ geometries, after which followed the development of TMD-based light-emitting diodes, photodiodes and solar cells. In 2015, near-unity photon quantum yield had been demonstrated in TMD monolayer^39^ which led to the development of highly efficient optoelectronic devices. TMDs and related vdW heterostructures possess weak dielectric screening and strong geometrical confinement which give rise to an extraordinarily strong Coulomb interaction, presenting a new paradigm for fundamental exciton physics. It also opens the door for probing fascinating many-particle phenomena, such as formation of different types of excitons including optically allowed and forbidden dark excitons as well as spatially separated interlayer exciton states.^40–45^ They exhibit binding energies in the range of 0.5 eV, which is one to two orders of magnitude larger than in conventional materials, such as in GaAs.^7–10,46^ In these materials, excitonic features being very stable, dominate the optical response and non-equilibrium dynamics. Trions^10–12^ and biexcitons^13,14^ have also been observed in monolayer TMDs.

In 2010, Heinz^5^ and Wang^6^ first showed that MoS_2_ undergoes a transition from an indirect-gap semiconductor in the bulk case to a direct-gap material in monolayer case, resulting in a drastically enhanced PL. The research field of optics and optoelectronics in TMDs experienced a boom after this discovery. First principle calculations, by analyzing the orbital character of electronic states at relevant high symmetry points, show us that the observed transition can be understood as a consequence of momentum or orbital selective interlayer splitting of the relevant energy levels.^47^ In the optical spectra of TMDs, two strongly pronounced resonances denoted by A and B excitons arise.^5^ The origin of these peaks can be attributed to the strong spin–orbit coupling in these materials lifting the spin degeneracy of the valence and conduction band. The splitting in valence band levels can reach approximately 200 meV in Mo-based TMDs and even 400 meV in tungsten (W)-based TMDs.^48^ The two optical transitions therefore evolve from holes in the upper and lower spin valence bands.

The review article has been divided into four sections: Section 2 reviews the important role of dark exciton states in determining the degree of polarization of PL emission and providing a robust reservoir for valley polarization. Section 3 reviews the relaxation mechanism of electron–hole pairs upon optical excitation. Section 4 reviews the intrinsic and extrinsic mechanisms that control exciton dynamics, transport and annihilation in 2D TMDs. Intrinsically dark and bright exciton energy is likely to play a key role in modulating the dynamics. In extrinsic regime, defect scattering becomes dominant in single layer TMDs leading to rapid picosecond decay and restricts exciton transport. The exciton–exciton annihilation is also a dominant process in single layer TMDs playing an important role in non-radiative recombination rate in regime of high exciton densities. Section 5 reviews the studies in optical response after ultrafast excitation in monolayer TMDs. Femtosecond pump–probe spectroscopy has been widely used to track the transient changes of the optical response of TMD monolayers. The role of electronic and phononic excitation in optical response can be investigated by the interplay of many body interactions from photoexcited carriers and the subsequent transfer of excitation to the phonon system followed by the cooling of the material through heat transfer to the substrate. In Section 6, we provide an overview of excitonic transport based optoelectronic devices, such as electrically driven light emitters, opto-valleytronic devices and photovoltaic solar cells bearing in mind the prominent role of excitonic effects. We finally conclude giving a touch on the remaining challenges in this field.

## 2. Dark excitons and valley polarization in 2D TMDs

TMDs such as MoS_2_, MoSe_2_, WS_2_ and WSe_2_ in their bulk forms, are indirect band-gap semiconductors with conduction band minimum (CBM) located half way between K and Γ points of the Brillouin zone, while the valence band maximum (VBM) is located at the Γ point. In monolayer TMDs, the band edges are located at the K and K′ corners of the hexagonal Brillouin zone.^5,6,49,50^ These points are degenerate but non-equivalent. The non-zero magnetic moments in the valence band electrons in K and K′ valleys are related to hybridization of d_*x*^2^−*y*^2^_ and d_*xy*_ orbitals.^51^ In K and K′ valleys, breaking of symmetry (both inversion and time-reversal) usually lead to magnetic moments in opposite directions resulting in valley-dependent optical selection rules. K and K′ valleys can be selectively excited by *σ*^±^ circularly polarized photons. It is well known that spin–orbit interaction in TMDs lifts the degeneracy of spin states in valence band. The observations of A and B excitonic transitions in the reflectance/absorption spectra are a result of this lifting of spin degeneracy.^5,9,52,53^ Spin–orbit interaction, in combination with broken time reversal symmetry is responsible for coupling of spin states and valley indices, making them ideal systems for valleytronics.^15,16,54,55^ Spin states are usually reversed in K and K′ valleys.^15^

**Fig. 1:**
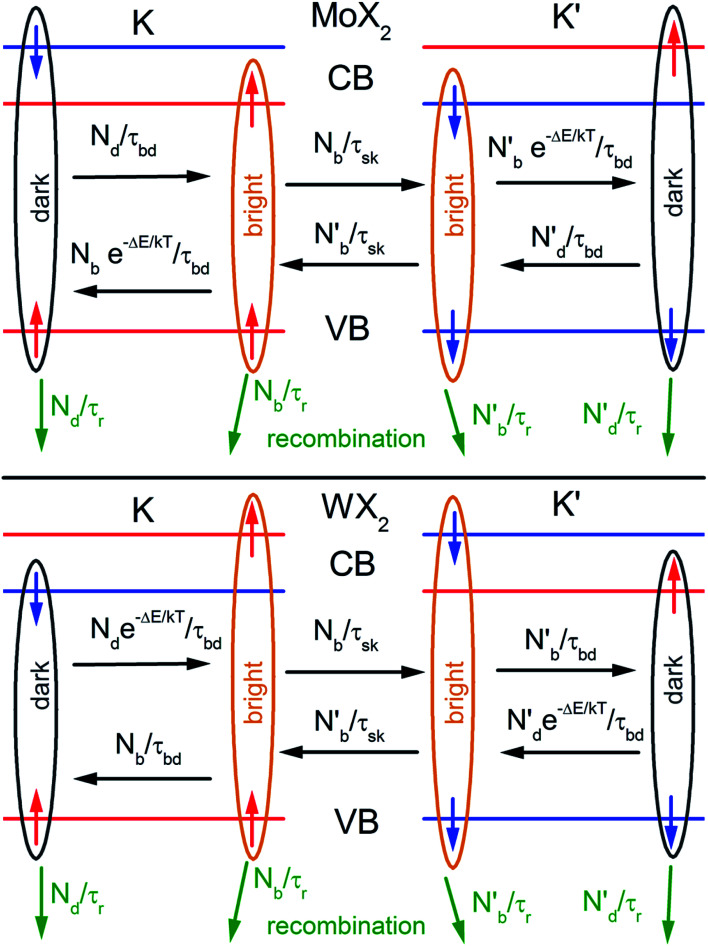
Schematic showing the band structure and spin configuration of the bright and dark A-excitons in MoX_2_ and WX_2_ materials. The possible inter and intra valley scattering and recombination paths included in the rate equations (see text) are indicated schematically. The influence of B-excitons can safely be neglected due to the large spin–orbit splitting in the valence band. (Adapted with permission from ref. 64 Copyright 2017 IOP Publishing).

The spin–orbit interaction in the conduction band, which typically is in the range of few to tens of meV,^49,56^ lifts the degeneracy of the bright and dark exciton states of A- and B-excitons. The spin configurations of A excitons in MoX_2_ and WX_2_ (X = S, Se) are shown in Fig. 1. Based on theoretical calculations^49,51,56^ and experiments,^57,58^ it is concluded that Mo and W-based TMDs have bright and dark exciton ground states respectively due to reversal of spins in the conduction band. In such TMDs, robust valley polarization can be achieved because scattering between K and K′ valleys is suppressed due to spin valley locking and large distance in *k* space between K and K′ valleys and significant valence band splitting. Hence a significant degree of circular polarization of PL emission can be expected in Mo- and W-based TMDs when excited by circularly polarized light.^17,19,59–62^ The K and K′ valleys can be selectively excited by *σ*^±^ circularly polarized photons, respectively.^63^ Polarization resolved PL shows that valley polarization persists for a sufficiently long time to be observed.

In TMD W- and Mo-based monolayers, the degree of circular polarization can be directly related to the alignment of dark and bright exciton states. A rate equation model has been proposed^64^ (Baranowski *et al.*) that demonstrates that different alignment of excitonic states that can enhance or suppress the circular polarization in PL emission, affecting the valley polarization. The kinetics of valley polarization can be explained by the two coupled rate equations:1
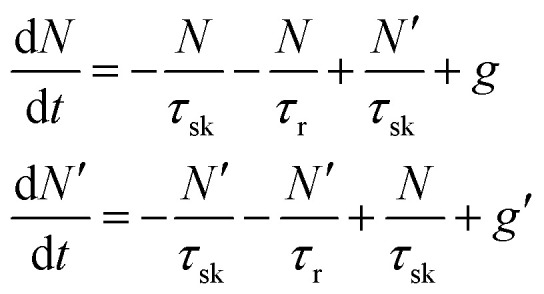
where *N* and *N*′ are the exciton populations in the K and K′ valleys, *τ*_sk_ is the inter-valley (exchange) scattering time (of bright excitons), *τ*_r_ is the recombination time of excitons (both radiative and non-radiative recombination), *g* and *g*′ being the generation rate of excitons in K and K′ valleys. The degree of polarization *P*(*t*) is defined by:2
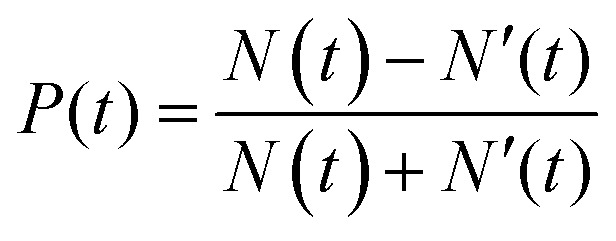


To include the effect of scattering between bright and dark states, four coupled rate equations are required. The system of rate equations for WX_2_ materials are as follows:3
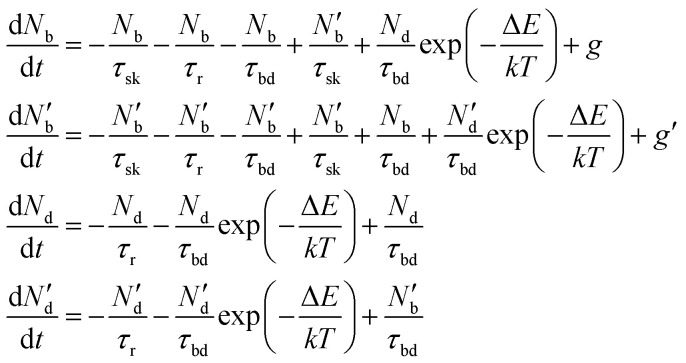
and for MX_2_, it has the following form4
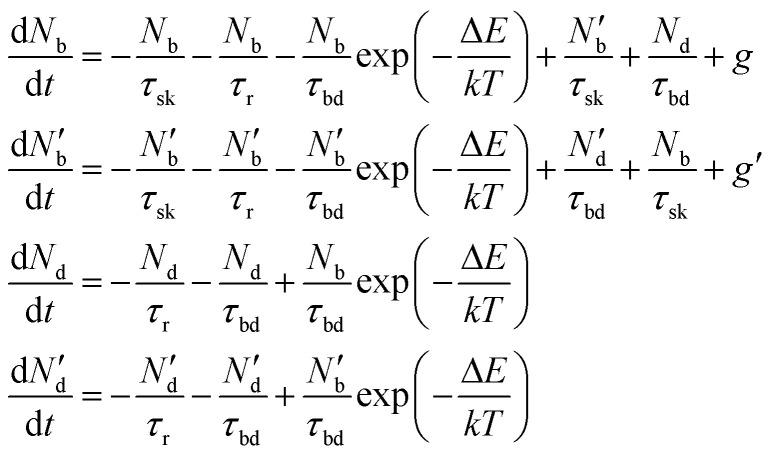


The inter- and intra-valley recombination paths involved in above equations are summarized in Fig. 1. Under steady state conditions 
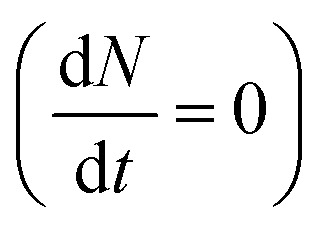
 and considering only dark–bright exciton scattering in the rate equations, we obtain for WX_2_,5
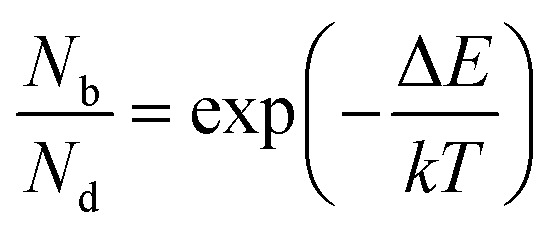
and for MoX_2_,6
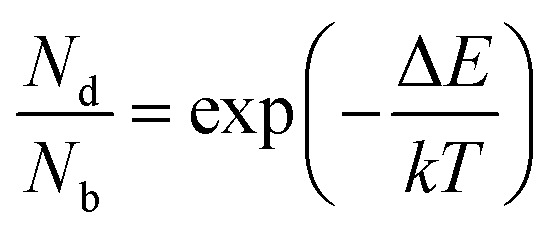
which are simply the Boltzmann distribution. Thus when the dark–bright scattering time (*τ*_bd_) is similar or shorter than the recombination time (*τ*_r_) (which is actually the case^65–67^ in TMDs *τ*_bd_ ≪ 1 ps and *τ*_r_ is in the range of pico seconds to hundreds of picoseconds^39,57,58,68–73^) and the dark exciton is the ground state (WX_2_ TMDs), it provides an important reservoir for valley polarization. In the situation of a valley polarization, the bright exciton population is always maintained at Boltzmann distribution in the same valley. As there exist no inter valley scattering channel for dark excitons,^74^ this reservoir of dark excitons being in ground state is a robust one.

The degree of valley polarization, related to occupation of excitons in *bright* state in K and K′ valleys, is given by7
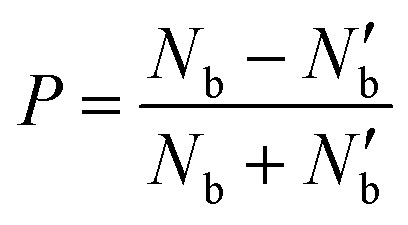


The above rate equations are solved to obtain the polarization assuming that excitons are generated only in the K valley corresponding to *σ*^+^ excitation (*g*′ = 0). The generation rate is assumed to be constant (cw excitation) and polarization at steady state conditions 
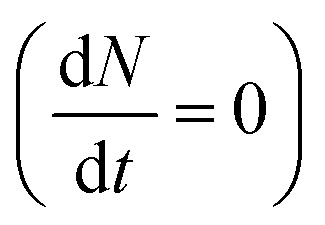
 is calculated.

**Fig. 2:**
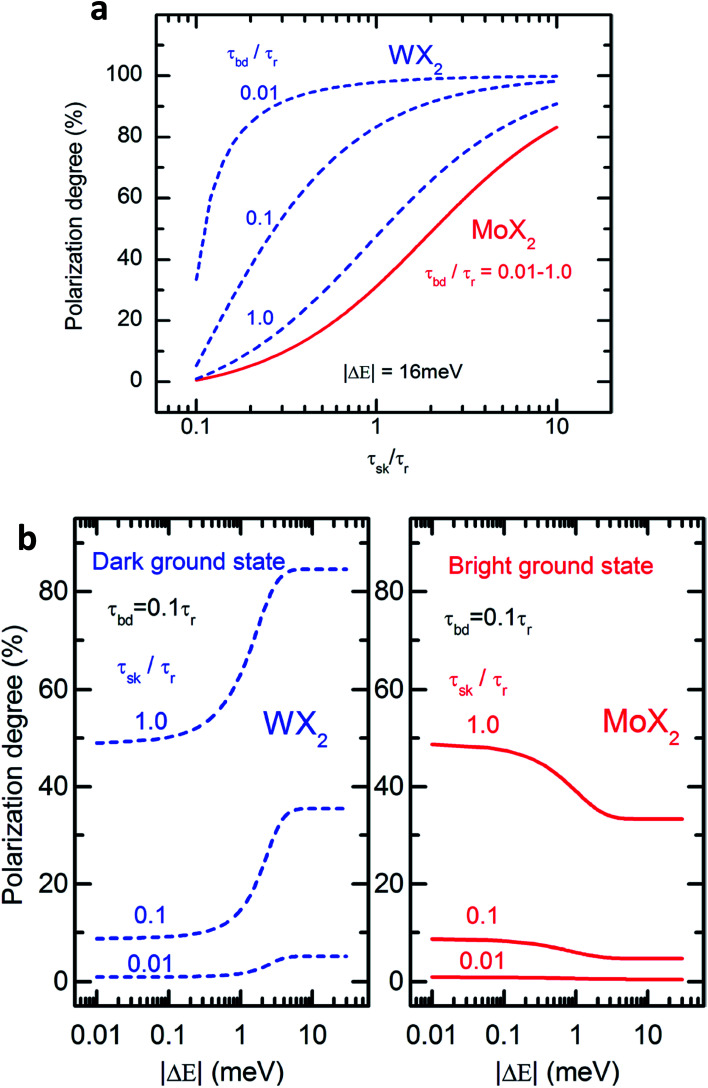
(a) Degree of circular polarization in PL as a function of the inter-valley scattering time calculated using the rate equations for WX_2_ (broken lines) and MoX_2_ (solid lines) for three different values of the bright–dark exciton scattering time *τ*_bd_. (b) Dependence of PL circular polarization degree as function of absolute value of bright–dark exciton spin splitting calculated according to rate eqn (3) (WX_2_) and eqn (4) (MoX_2_) for different values of inter-valley scattering time. (Adapted with permission from ref. 64 Copyright 2017 IOP Publishing).

Fig. 2(a) shows the degree of polarization in PL emission as functions of intervalley scattering time for WX_2_ and MoX_2_ TMDs. It has to be remembered that excitons are always *created* in bright state. They can either be scattered in the same bright state in the neighboring valley (intervalley scattering)^75–77^ or to the dark state in the same valley (intravalley scattering). Clearly the degree of polarization decreases with decrease of intervalley scattering time. This is expected as decrease in *τ*_sk_ would mean an increase in *N*′_b_ decreasing *P*. Also noticeable is the fact that TMDs with dark exciton as the ground state (WX_2_) exhibit larger degrees of polarization than those having bright exciton ground state (MoX_2_). This is because in WX_2_, there is a reservoir of dark excitons created which always tries to maintain the 
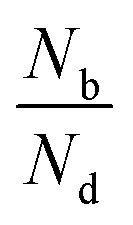
 ratio close to the Boltzmann distribution, thus increasing the degree of polarization of PL emission (and also the total valley polarization). In contrary, the intravalley bright–dark scattering in MoX_2_ TMDs is damped inhibiting the degree of polarization of PL emission (together with valley polarization). On the other hand for MoX_2_ TMDs, changes in dark–bright scattering time do not seem to affect the polarization. This indicates clearly that intervalley scattering play a dominant role in the exciton kinetics for such systems. Also noticeable is that for very efficient dark–bright intravalley scattering (*τ*_bd_ ≪ 1), the polarization dependence exhibits a plateau for decent intervalley bright exciton scattering
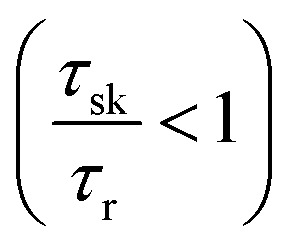
. This result agrees well with experiments carried out in WX_2_ TMDs,^74^ where the polarization dependence does not change much with excitation energy (exciton scattering rate increases with increase in excitation energy).

The polarization dependence on dark–bright splitting energy is also different for two systems, having ground state as dark exciton (WX_2_) and bright exciton (MoX_2_) (Fig. 2(b)). When the dark and bright exciton states are degenerate (Δ*E* ≪ *kT*), there is no difference in the degree of polarization in either system. When the splitting between the bright and dark states become non-negligible, the degree of polarization is much higher for WX_2_ TMDs (as compared to MoX_2_ TMDs) because of an increase in population of dark excitons. In contrary for MoX_2_ TMDs, increase in splitting energy decreases the population of the excitons in the dark state, thus decreasing the degree of polarization.

It is intriguing that despite the large valence band splitting (due to large spin–orbit interaction) and spin valley locking, intervalley scattering is still effective in TMDs and valley depolarization occurs in the range of few to several picoseconds.^78–82^ One proposition in literature explaining exciton scattering between valleys is the two longitudinal acoustic (LA) phonon model. The model explains thermalization of electron–hole pairs *via* the emission of two LA phonons.^16,75,77^ Excitons can get scattered between K and K′ valleys provided the exciton kinetic energy is above the energy of 2 LA phonons at the K point. The LA phonon model provides a relatively straightforward way to describe the degree of PL circular polarization as functions of excitation energy, but had its own shortcomings. Alternative explanations for valley depolarization has also been credited to valley electron–hole exchange interaction^74,83^ – a mechanism in which there is a virtual recombination of a bright exciton in the K valley and the creation of a bright exciton in the K′ valley. This scattering is faster when exciton kinetic energy is higher and explains the dependence of degree of circular polarization of PL emission on excitation energy.

## 3. Photocarrier relaxation pathways in 2D TMDs

**Fig. 3:**
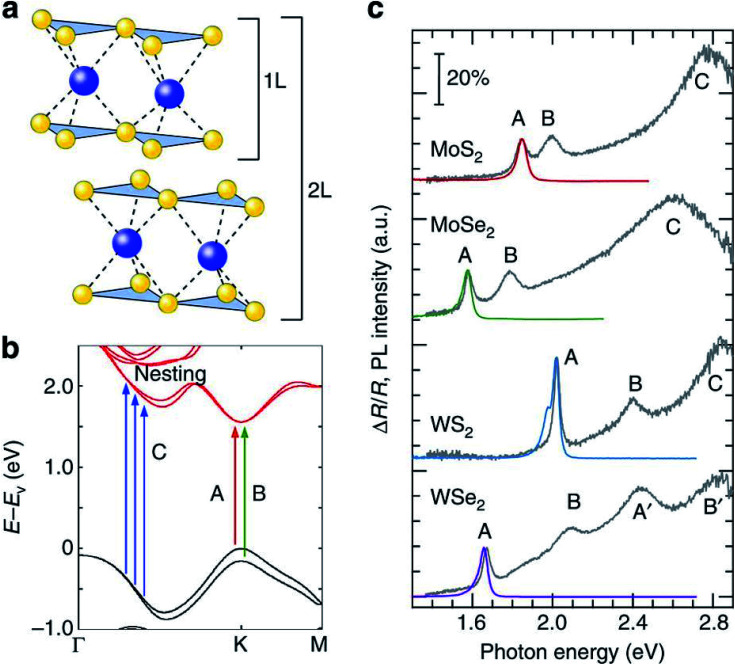
Properties of monolayer MX_2_. (a) Lattice structures of monolayer and bilayer MX_2_. (b) The band structure of monolayer MoS_2_ with the label of C calculated by the DFT. The arrows indicate the transition in A, B and the band nesting (C). (c) PL spectra (red, green, blue and purple curves) from excitation at the C (A′ for WSe_2_) peak and differential reflectance spectra (grey curves) of monolayer MX_2_ flakes on quartz substrates. The scale bar indicates 20% absorption based on the differential reflectance spectra. The PL intensity is normalized by the A exciton peak of the differential reflectance spectra for each material and the spectra are displaced along the vertical axis for clarity. (Adapted with permission from ref. 92 Copyright 2014 Macmillan Publishers).

The TMD layers, despite being atomically thin, are excellent absorbers of light.^84–86^ Typical absorption spectra, consisting of several characteristic peaks due to exciton resonance and interband transitions, have been depicted in Fig. (3). Theoretical studies for such systems have reported the extraordinary light–matter interaction to a very special feature in the density of states, *i.e.* ‘band nesting’. This leads to singularity features in the joint density of states.^87^ The conduction and valence bands are parallel to each other in the band nesting region, diverging at resonance energy. This results in a giant enhancement in the corresponding optical conductivity, a characteristic feature in low dimensional systems.^88^ For photon energies corresponding to transitions between van Hove singularity peaks in density of states in MX_2_ compounds, absorption is found to be strongly enhanced. Such peaks in the DOS arise due to heavy effective carrier masses in MX_2_ compounds.^88,89^ Because the absorption is highly efficient under resonance conditions, it is important to study the photocarrier relaxation dynamics after excitation in order to exploit these materials into light-harvesting devices. In particular, photocarriers generated in the band nesting region are of particular interest as the electrons and holes are expected to relax at the same rate, but with opposite momentum. For monolayer MX_2_, the conduction band minimum (CBM) and valence band maximum (VBM) are both located at K/K′ point of the Brillouin zone. The conduction band valley at Λ point and the valence band hill at Γ point play a dominant role in the direct to indirect band gap crossover. The location of band nesting region is near the midway between Λ and Γ points.

Fig. 3(c) shows the PL emission and differential reflectance spectra of monolayer MoS_2_, MoSe_2_, WS_2_, and WSe_2_ on a quartz substrate. Apart from resonance peaks A and B corresponding to the excitonic transitions occurring at K/K′ points,^90,91^ there is a strong absorption at higher energies (C peak for MoS_2_, WS_2_ and MoSe_2_) estimated to be above 30% based on reflectance and transmittance measurements. This feature corresponds to the region of Brillouin zone where valence and conduction bands are nested.^87,92^ Band structure calculations in TMDs indicate that excited electrons and holes in the nesting region relax to their immediate band extremum, corresponding to Λ valley for electrons and Γ hill for holes. In monolayer and few layer MoS_2_, this intraband relaxation is extremely fast (<500 fs) and dominates other relaxation processes.^93–95^ The electron–hole pairs separated in *k*-space requires emission or absorption of a phonon, and as such, the radiative recombination of these pairs is a slow process. This results in a low yield emission in PL. It is to be noted that carrier lifetimes of direct excitons are of the order of 100 ps with high quantum yield (QY)^93^ as compared to those in indirect emission process which can be estimated to be on the order of 1 ns.^93,96^

**Fig. 4:**
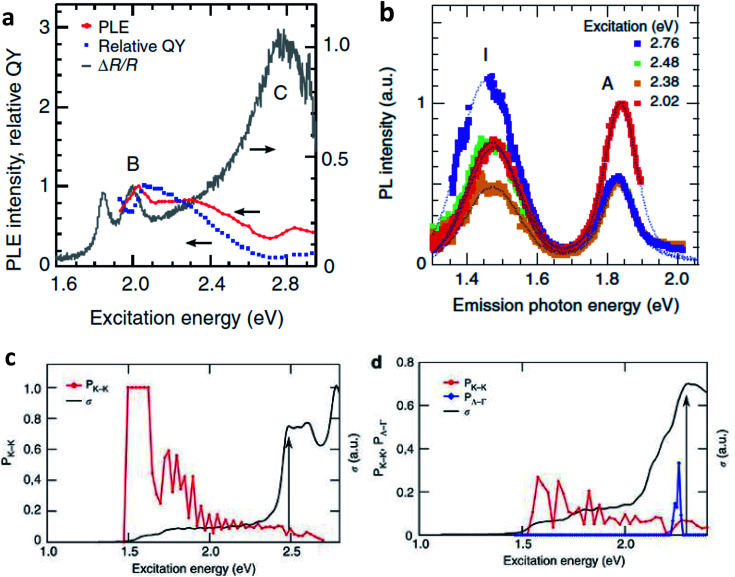
(a) PLE spectra and relative QY of emission for band gap emission for monolayer MoS_2_ flakes. Differential reflectance spectra are also shown for comparison. The PLE spectra are based on the integrated intensity of the A peak in the PL spectra at each excitation energy. The PLE spectrum is normalized by the B exciton peak of the material. (b) PL spectra of bilayer MoS_2_ flake collected with excitation energy of 2.02, 2.38, 2.48, 2.76 eV. (c, d) The fraction of electron–hole pairs that end the relaxation at the K point (*P*_K_K_, red curve) and the optical conductivity (*σ*, black curve) for monolayer (c) and bilayer MoS_2_ (d). For (d), the fraction of electron–hole pairs relaxing to Λ valley and Γ hill (*P*_Λ_Γ_) is also shown (blue plot). The black arrows indicate the position of the first peak due to band nesting. (Adapted with permission from ref. 92 Copyright 2014 Macmillan Publishers).

Excitation dependent photoluminescence (PLE) studies can be performed to observe the effect of band nesting on the photoexcited electron hole pairs. Fig. 4(a) show comparisons between PLE spectra (red plots), relative QY of emission (blue dots), and the differential reflectance spectra (grey lines) of monolayer MoS_2_. The PLE intensity is enhanced when the excitation is in resonance with B exciton. In contrast, there is a suppression of PLE intensity at the excitation near C exciton absorption. The relative QY drops when the excitation is above B exciton resonance. The suppression of relative QY near C exciton absorption can be attributed to instantaneous separation of electron hole pairs separated in *k*-space due to band nesting.

The PLE and absorption spectra of bilayer MoS_2_ are also similar to that of monolayer MoS_2_, exhibiting A and B exciton resonance and strong C peak absorption due to band nesting. Studies^97,98^ show that bilayer MoS_2_ and WS_2_ exhibit indirect band gap emission involving Λ valley in conduction band and Γ hill in valence band. It is clear that an enhancement in band gap indirect emission is expected when excited in the band nesting region. The PL spectrum of the indirect emission peak (I) exhibits remarkable enhancement over direct emission peak (A) as excitation energy approaches the band nesting energy (Fig. 4(b)). It may also be noted that QY of MoS_2_ bilayer samples is distinctly smaller than that of the monolayer one near B peak excitation. The results point out to the fact that photocarrier relaxation pathways and radiative emission channels are uniquely related to band nesting.

The photocarriers relax through a series of phonon scattering events – both acoustic and optical phonons can intervene the relaxation. The carrier relaxation is subjected to selection rules imposed by energy and momentum conservation. The electron–phonon scattering time, under the approximation of weakly interacting electron and hole, is given by Fermi's golden rule.^99,100^8
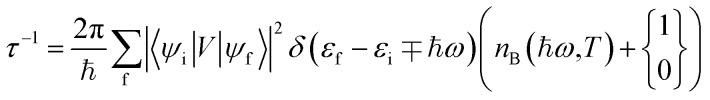
where *ψ*_i_〉 and *ψ*_f_〉 are the initial and final states with momentum *k*_i_ and *k*_f_ = *k*_i_ − ***q*** and energies *ε*_i_ and *ε*_f_. ***q*** is the scattering vector, *V* is the interaction potential, and *n*_B_(*ℏω*,*T*) is the Bose–Einstein distribution. The upper and lower elements correspond to phonon emission/absorption respectively. According to DFT results, majority of photocarriers (electrons and holes) are likely to relax into Λ and Γ points respectively when excited in the band nesting region.^92^

Fig. 4(c) and (d) show the fraction of electron–hole pairs that end the relaxation at K point as a function of excitation energy for monolayer and bilayer MoS_2_ respectively. The probability of electron–hole pairs relaxing to K is unity, when the excitation energies are between A and B peaks. This fraction becomes considerably lower at energies close to the band nesting region ∼2.0 eV. The increase in optical conductivity is also high at band nesting energies. This trend explains well the decrease of relative quantum yield at the C absorption peak in monolayer MoS_2_ (Fig. 4(a)), and from this energy onwards, the relaxation is mostly mediated by acoustic phonons. For bilayer MoS_2_, the fraction of photocarriers relaxing to the K point decreases and there is a significant enhancement in the fraction of carriers relaxing to Λ valley and Γ hill. This further explains the enhancement of the indirect emission peak in these materials (Fig. 4(b)) near C peak absorption. Note also the corresponding increase in optical conductivity in the band nesting region, where the population of electron–hole pairs at different momentum spaces are already high.

**Fig. 5:**
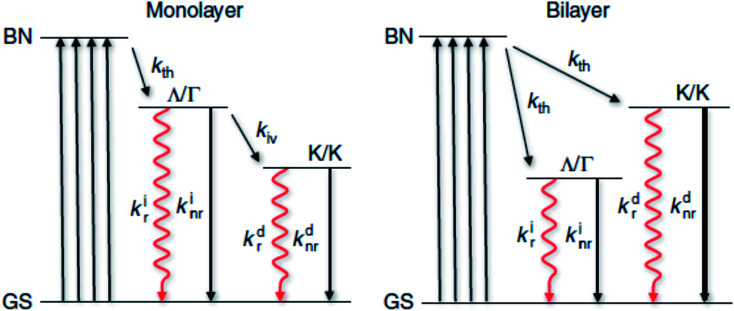
Excitation and relaxation pathways for photocarriers. Energy diagram representing photocarrier relaxation channels in monolayer and bilayer MX_2_ where the initial excitation is from the ground state (GS) to the band nesting (BN) energy. Nonradiative transition is indicated with a black solid arrow. A rate constant *k* is associated with each transition. The subscripts indicate the types of transition: intravalley thermalization (*k*_th_), intervalley scattering (*k*_iv_), radiative (*k*_r_) and nonradiative (*k*_nr_). The superscripts (i) and (d) indicate indirect and direct transitions, respectively.

Based on these investigations, the possible relaxation pathways for monolayer and bilayer MX_2_ can be summarized as shown in Fig. (5). When the system is initially excited from the ground state to the band nesting (BN) excited state, a large fraction of these excited states relaxes to another excited state (Λ/Γ) representing the state where electrons occupy the Λ valley and holes occupy the Γ hill. Radiative recombination from this state competes unfavorably with the non-radiative decay (usually a fast process, 2–4 ps)^93^ and intervalley scattering (*k*_iv_) to the lowest excited state (K/K) where both electrons and holes occupy the K point. Hence, only a small fraction of the excited states are transferred to the K/K states where radiative decay occurs with a modest yield. In bilayer MX_2_, there is a sizeable fraction of the excited states that can decay into the indirect (Λ/Γ) state where radiative ‘indirect’ emission occurs with a modest yield. This indirect emission does not compete with direct emission, as is the case for a monolayer. Also possible is the occurrence of hot electron emission from the K/K′ state with non-negligible efficiency, partly due to intraband relaxation and intervalley scattering of hot carriers. Band nesting plays a crucial role in the spontaneous separation of electron–hole pairs in the *k*-space and temporary suppression of their relaxation to the fundamental band edge.

## 4. Defect assisted recombination in exciton dynamics and transport in 2D TMDs

**Fig. 6:**
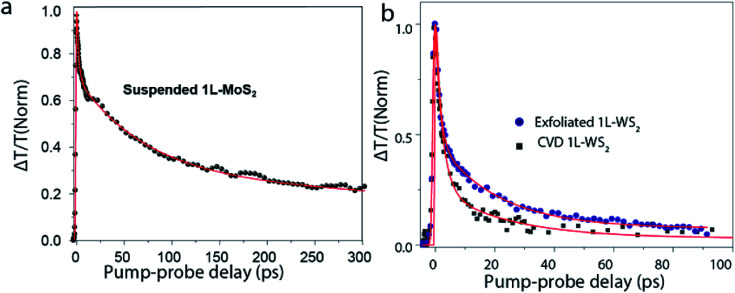
(a) TA dynamics of a suspended exfoliated 1L-MoS_2_ flake. Red line is a fit using a triexponential function convoluted with an experimental response function. Pump fluence is 0.6 μJ cm^−2^. (b) TA dynamics of exfoliated 1L-WS_2_ on SiO_2_ substrate and CVD 1L-WS_2_ on sapphire. Red lines are fits using a biexponential function convoluted with an experimental response function. Pump fluence is 1 μJ cm^−2^. (Adapted with permission from ref. 104 Copyright 2017 ACS Publications).

Defects are one of the primary sources of external environmental factors that contribute significantly to the non-radiative pathways.^101^ In monolayer TMDs, non-radiative rather than radiative relaxation pathways dominate the exciton dynamics. Many research groups were successful in improving PL quantum yield by surface passivation techniques^93,102,103^ pointing to the important role of defects in exciton dynamics. Fig. 6(a) shows the transient absorption decay of A-exciton in a suspended MoS_2_ monolayer^93,104^ displaying multi-exponential behavior, implying that more than one relaxation processes are involved. The fast picosecond decay is attributed to the defect related recombination. It turns out that for monolayer TMDs, the number of surface and interfacial defects are greatly enhanced as compared to thick crystals, as almost all the atoms are on the surface or at interfaces. This increases the probability of defect scattering processes in these monolayers. Recently, defect assisted recombination has been investigated by Wang *et al.*^69^ and concluded that there are two stages of recombination – a fast pathway with a time scale of 1–2 ps, and a slower one in a time scale of 60–70 ps. Defect related relaxation pathways lead to the variation of exciton dynamics for samples fabricated by different methods. Fig. 6(b) shows the transient absorption (TA) dynamics for 1L-WS_2_ grown by two different methods-mechanical exfoliation and chemical vapor deposition (CVD). Initial exciton decay, up to a time scale of 5 ps is similar for both the samples indicating relaxation to the dark exciton state is the dominant process here. At a time scale >5 ps, exciton decay is faster for CVD grown sample as compared to the mechanically exfoliated one signifying that defect related mechanism appear to be dominant. This is consistent with the fact that defect density in CVD grown WS_2_ (∼3 × 10^13^ cm^−2^)^105,106^ is four orders or magnitude higher than that of exfoliated WS_2_ (∼2 × 10^9^ cm^−2^).^106^

**Fig. 7:**
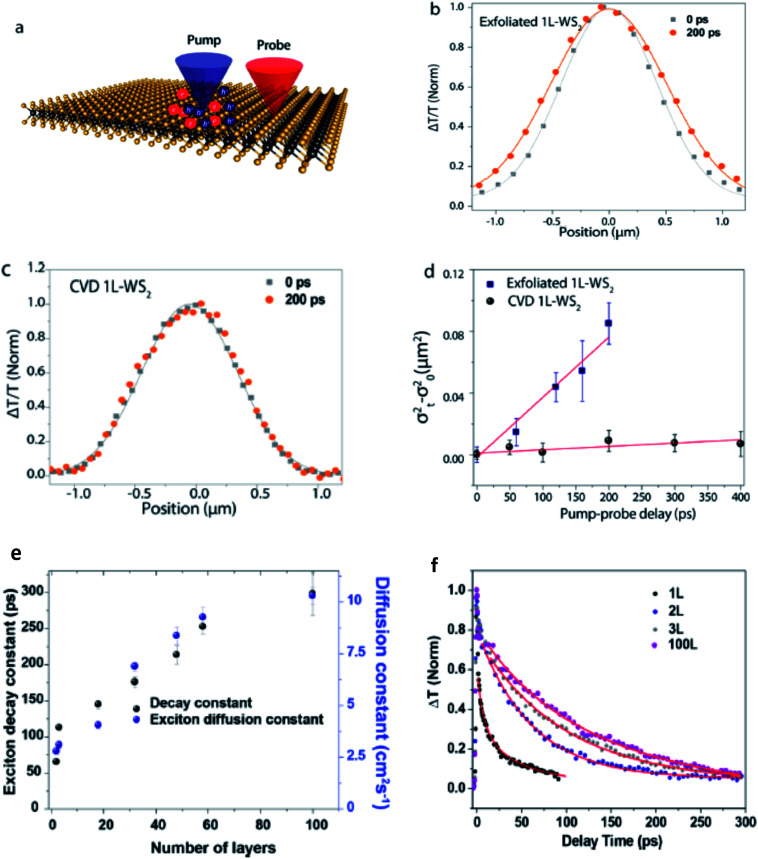
(a) Schematic description of the exciton diffusion measurements. Spatial profiles of excitons in an exfoliated 1L-WS_2_ (b) and a CVD 1L-WS_2_ (c) at different pump–probe delay times. (d) Diffusion constants are obtained from the linear fitting of the variance of Gaussian profiles using eqn (12). Red lines are the linear fits. (e) Extracted decay constants and exciton diffusion constants are plotted as a function of number of layers. (f) Thickness-dependent exciton dynamics are modeled by eqn (10). (Adapted with permission from ref. 104 Copyright 2017 ACS Publications).

Exciton–phonon scattering, an intrinsic effect,^107^ together with extrinsic factors such as defects and impurities, sets the upper limit of exciton mobility. Transient absorption microscopy (TAM) can be used to map the exciton population in both spatial and temporal domains.^108,109^ In this process, the pump is kept at a fixed position and the probe is scanned relative to the pump by two galvanometer scanners. The pump induced change in transmission Δ*T* = *T*_pump-on_ − *T*_pump-off_ is then collected. At zero delay time, TAM reflects the initial population excitons created by the pump beam. At a later delay time, TAM images visualize how excitons transport out of the initial volume. The exciton transport is treated as in-plane 2D diffusion, since the transient absorption signal is integrated over the *z*-direction (perpendicular to the plane). The anisotropy of 2D exciton propagation can be neglected, meaning exciton diffusion along *x* and *y* directions are independent and proceed with identical rates. Fig. 7(b) and (c) show the one dimensional exciton profile of the exfoliated 1L-WS_2_ and CVD grown 1L-WS_2_. The initial population *n*(*x*,0) following a Gaussian distribution as created by the Gaussian pump beam at *x*_0_ is given by9
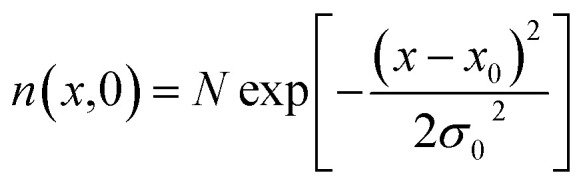
with a variance of *σ*_0_^2^. This initial population is almost exclusively in the bright state as because the oscillator strength of the dark state is negligible.

Exciton population as a function of space and time can be described by a differential equation that describes the diffusion out of the initial volume, and the population decay which is given by10
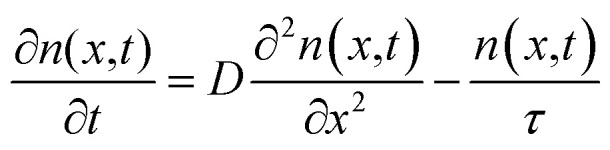
here *D* is the diffusion constant and *τ* represents the exciton lifetime that includes the contribution from both bright and dark states. The solution of the above equation can be given as11
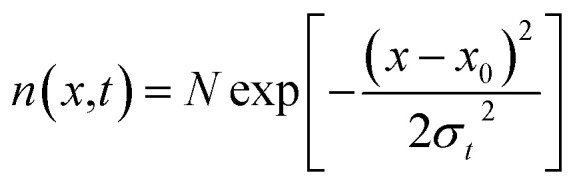


This implies that the exciton density at a later time ‘*t*’ is also Gaussian with a variance *σ*_*t*_^2^. The diffusion constant is given by12
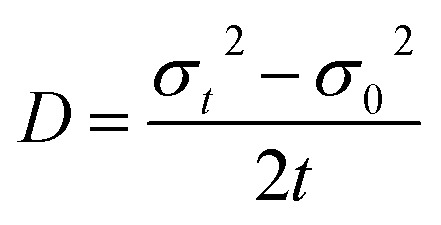
where ‘*t*’ represent the thickness of the film. (Typical values of exciton diffusion constant of exfoliated and CVD grown WS_2_ are 2 cm^2^ s^−1^ and 0.1 cm^2^ s^−1^ respectively). Whereas exciton transport is severely impeded in CVD grown WS_2_, long range motion of exciton over time scale of 100 ps can be seen in exfoliated grown WS_2_ (Fig. 7(b)). This is consistent with high defect density found in CVD grown WS_2_ (∼3 × 10^13^ cm^−2^) than in exfoliated WS_2_ (∼2 × 10^9^ cm^−2^).^106^ The drastic difference in exciton dynamics are clearly governed by defect and/or impurity scattering for CVD grown sample.

At the limit of single layer, excitons are subjected to interfacial Coulomb scattering by charged impurities^110–112^ and scattering from substrate phonons in addition to intrinsic phonon scattering. Exfoliated WS_2_ layers with thickness ranging from 1 layer to 100 layers have been investigated for measuring their mobility in order to reach the intrinsic limit.^104^ Usually charged impurities and surface optical phonons are negligible as number of layers increase. Previous electron mobility measurements of WS_2_ layers showed a maximum value of 240 cm^2^ V^−1^ s^−1^ at a thickness of 10 nm.^110,113^ Therefore exciton mobility increases as the thickness increase until we reach a stage where only intrinsic factors can limit this mobility.

Fig. 7(e) shows that the diffusion constant in WS_2_ monolayer increases monotonically with the number of layers due to the reduction in defect scattering. The exciton diffusion constant of 100L-WS_2_ is 10.4 ± 0.4 cm^2^ V^−1^ s^−1^, about 5 times higher than that in the 1L-WS_2_. As the thickness of the layer increases from 1L to 2L, there is a transition from a direct to an indirect exciton, changing the exciton dynamics completely. As thickness increases to more than 2L, the conduction band minimum moves to the Λ point, while the valence band maximum remains at the K point, leading to the formation of indirect exciton (I). The slower exciton decay in 2L than in 1L can be understood as the relaxation of an indirect exciton that requires intervalley and interband phonon scattering. Thus, exciton dynamics for 2L and thicker are ascribed to the intervalley and interband phonon scattering. Fig. 7(e) also reveals that when the thickness of layers becomes larger than 20L, exciton mobility (a measure of diffusion constant) tracks the exciton decay time almost perfectly. Because the indirect exciton decay time (greater than 2L) directly reflects exciton–phonon scattering time, Fig. 7(e) implies phonon-limited exciton transport is achieved in layer greater than 20. It is important to note here that some of these results do contradict with results obtained for thickness dependent exciton transport in 2d TMDs: for MoSe_2_ the exciton diffusion constant is lower for a single layer than for bulk^114^ whereas for WSe_2_ the exciton diffusion constant is higher for a single layer than for bulk.^115^

### 4.1 Non-radiative recombination due to exciton–exciton annihilation

A significant feature of low dimensional electronic systems is the enhanced many body interactions due to reduced dimensions.^116^ Many body scattering processes such as Auger recombination and exciton–exciton annihilation play a major role in non-radiative relaxation when the density of excitons is very high. Such non-radiative relaxation processes determine the upper limit of excitation density that affects the performance in device applications like semiconductor lasers, light-emitting diodes, *etc.* Exciton–exciton annihilation has been investigated in single layer, bilayer and trilayer WS_2_ by time-resolved PL spectroscopy.^117^ Initially, in the regime of low excitation density, a single exponential behavior emerges in PL dynamics. But when the excitation density is high (corresponding to higher pump intensities), a faster process due to exciton–exciton annihilation becomes prevalent. A rate equation, consisting of an exciton recombination and exciton–exciton annihilation term can be used to describe this process:13
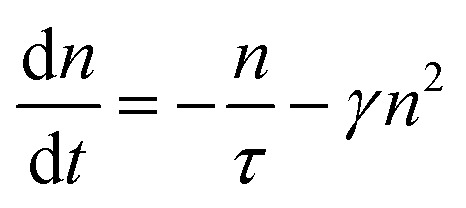
where ‘*n*’ is the exciton population, *τ* represents exciton lifetime without annihilation and *γ* represents the annihilation rate constant. Clearly annihilation processes are dominant in high ‘*n*’ regime. The solution of the above equation, considering time-independent nature of *γ*, can be given by14
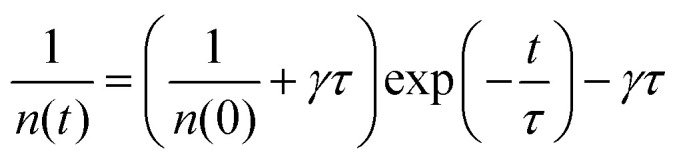
where *n*(0) is the initial exciton density.

PL dynamics at different excitation densities is used to extract exciton–exciton annihilation rate constants (*γ*). Typical values of *γ* obtained in 1L, 2L and 3L WS2 are 0.41 ± 0.02, 6.0 ± 1.1 × 10^−3^, 1.88 ± 0.41 × 10^−3^ cm^2^ s^−1^ respectively.^104^ The most significant result is the high annihilation rate constant in 1L WS_2_, almost 100 times more than 2L and 3L WS_2_. For1L-WS_2_, exciton–exciton annihilation occurs at exciton densities as low as 1 exciton per 10^5^ nm^2^. This corresponds to an averaged inter-exciton distance >600 nm whereas the spatial extent of an exciton in 1L-WS_2_ is roughly about 2 nm.^118^ The large inter-exciton distance implies that exciton diffusion process has to occur before annihilation. The two rate determining steps for describing the kinetics are:

(i) Diffusion of two excitons towards each other, and (ii) annihilation of two excitons when they are sufficiently close to each other.15
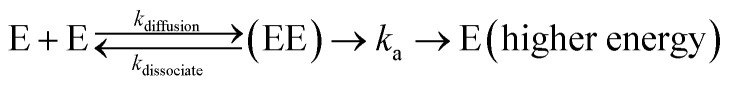
here E represents an isolated exciton and (EE) the exciton pair sufficiently close so that annihilation takes place. *k*_diffusion_ represents rate of change in number of close pair per unit area and *k*_a_ is the annihilation rate proceeding from (EE). The overall exciton–exciton annihilation rate becomes16
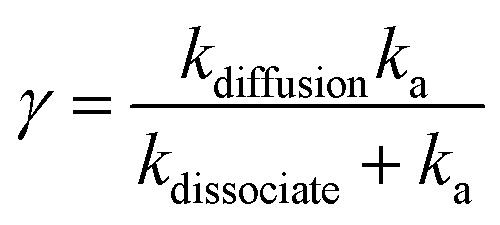


The exciton diffusion rate is typically an order of magnitude faster than overall exciton–exciton annihilation rate (*k*_diffusion_ ∼ *k*_dissociate_ ≫ *k*_a_). Exciton diffusion rate is higher in 2L- and 3L-WS_2_ than in 1L-WS_2_.

The thickness dependence of annihilation rate constant (*k*_a_) can be explained by considering that in the single layer limit, stronger Coulomb interaction leads to stronger many body interactions. It is well-known that dark excitons contribute significantly to the PL dynamics for 1L-WS_2_. Recent theoretical calculations show that Auger recombination can be very efficient out of the dark states.^119^ Furthermore, exciton–exciton annihilation requires conservation of energy and momentum. 2L- and 3L-WS_2_ are indirect semiconductors and momentum conservation requires assistance from phonons. Direct band-gap monolayer, on the contrary, does not require phonon assistance for conservation of momentum, making this process a much probable event, leading to at least, two orders of magnitude larger values of *γ*, as compared to bilayer and trilayer WS_2_. In other words, in bilayer and trilayer WS_2_, only a fraction of exciton encounters results in annihilation.

Other single layered TMDs exhibit similar exciton–exciton annihilation rate.^120–122^ In 1L-MoSe_2_, Kumar *et al.*^122^ measured the annihilation rate to be 0.33 cm^2^ s^−1^ whereas for 1L-WS_2_ (Mouri *et al.*^120^), it was found to be 0.36 cm^2^ s^−1^. Compared to 2D quantum well nanostructures,^123^ the exciton–exciton annihilation rates were found one order higher in 2D TMDs. The efficient exciton–exciton annihilation in 1L-WS_2_ implies that the inverse process, that is, multiple exciton generation could also be effective.^124^ The exciton–exciton annihilation time in 1L-WS_2_ was found to be ∼400 ps (at an exciton density of 1.6 × 10^9^ cm^−2^) by using the same method used to obtain the Auger recombination time in quantum dots.^116^ Such slow exciton–exciton annihilation on hundreds of picoseconds time scale makes it quite possible to extract the additional exciton generated.

## 5. Optical response of monolayer TMDs after ultrafast excitation: role of electronic and phononic excitation

In order to understand how TMD systems respond to external perturbations, it is important to employ optical excitation and focusing particularly on the behavior of photoexcited charge carriers in these systems. Distinct phenomena, like photoexcited carrier lifetimes,^69,72,93,95,115,125,126^ exciton–exciton interactions and annihilation processes,^94,120–122^ coherent coupling and control,^127–129^ band gap renormalization,^44,130–132^ ultrafast structural deformation^133^ and spin valley dynamics^79–82,134–137^ have been explored. Ultrafast pump–probe spectroscopy is the most commonly used tool to study these phenomenon, where pump induced changes of optical response are investigated. Both photoexcited charge carriers and non-equilibrium phonon populations (including transient heating of lattice) can alter optical properties of the material. Typically in pump–probe spectroscopy, an analysis of evolution of exciton line shape is made after photoexcitation of TMD monolayer. Modifications of optical response are characterized by changes in the exciton peak area, line width and energy and their temporal variation.

The pump photon energy corresponding to a certain exciton resonance predominantly leads to optical injection of electron–hole pairs. Variation in pump fluence modify the electron–hole pair densities and can bring the system to the intermediate excitation regime, where interactions between charge carriers play a significant role. But the excitons may still remain in bound states, that is, their densities are below the so-called Mott threshold, where excitons dissociate. Optical excitation can thus cause changes in the charge state of monolayer samples through photodoping, together with creation of charged exciton states^10,11^ modifying the optical response.^11^

**Fig. 8:**
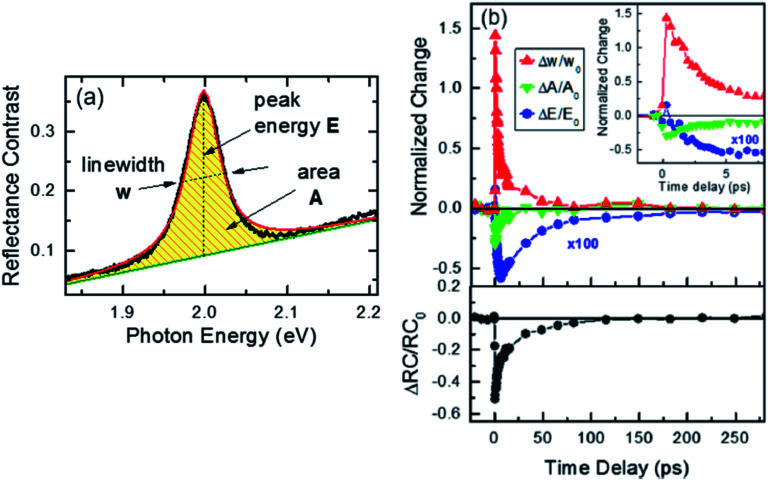
Quantitative analysis of the time-dependent line shape of the A exciton resonance in WS_2_ monolayer. (a) Representative reflectance contrast spectrum fitted with a Lorentzian line shape (red line) and linear offset (green line). (b) Relative changes of the line width Δ*w*/*w*_0_, area Δ*A*/*A*_0_, and resonance energy Δ*E*/*E*_0_ of the peak are presented as a function of time delay for a pump fluence of 101 μJ cm^−2^. The data are normalized to the corresponding values at negative delay times: *w*_0_ = 47 meV, *A*_0_ = 1 arb unit, and *E*_0_ = 1.996 eV. (inset) The relative change of the line width and area for short delay times. Lower panel: The relative change of the reflectance contrast RC at a probe energy of 1.997 eV, averaged over a narrow spectral range of 13 meV, is shown for comparison. (Adapted with permission from ref. 138 Copyright 2017 ACS Publications).

Fig. 8(a) depicts the line shape analysis of A exciton resonance of WS_2_ monolayer fitted to a Lorentzian function including a linear offset to account for the broad background. The reflectance contrast is defined as 
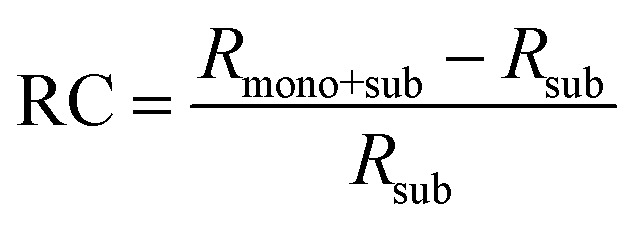
 where *R*_mono+sub_ and *R*_sub_ denote reflectance of WS_2_ monolayer on substrate and the bare substrate respectively.^138^ It is to be noted that for a transparent substrate covered by ultrathin layer producing a moderate RC signal, RC is determined by the imaginary part of dielectric function, that is, sample absorption. The pump induced changes in peak line width *w* (FWHM), the area *A*, and the resonance energy *E* can be monitored from the line shapes as functions of time delay after excitation. These parameters that are extracted from Fig. 8(a) are plotted separately in Fig. 8(b) corresponding to a pump fluence of 101 μJ cm^−2^. The change of the normalized reflectance contrast at the peak of resonance 
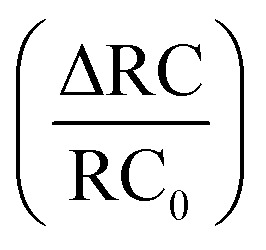
 (lower panel of Fig. 8(b)) and its temporal evolution reflects a complex combination of individual dynamics arising from peak shifts, broadening and decrease of peak area. Within the experimental resolution of several 100 s of picoseconds,^138^ changes in the optical response of WS_2_ monolayer appear immediately after pump excitation. A sharp increase of the line width of A exciton is initially observed followed by a simultaneous bleaching of resonance, that is, decrease in the area of the feature. The decay dynamics of the broadening and bleaching of resonance are in picosecond time scale. The peak area returns to its original value after a few 10 s of picoseconds, and the optical response mostly reflects a red shift of exciton transition energy. These changes then decay in 100 ps time scale, the system fully recovering after 250 ps. Both fast and slow dynamics that are observed in RC signal from WS_2_ monolayer can be characterized by temporal evolution of time constants.

At the excitation photon energy, the optical pulse creates electron–hole pairs and their subsequent scattering processes with phonons and with other carriers lead to rapid thermalization and relaxation of carrier population towards respective band minima. At exciton resonance, presence of photoexcited carriers in the material strongly modifies optical response with several distinct physical processes contributing to this response.^44,137,139^ Firstly, spectral broadening of the exciton peak can be caused by Coulomb scattering of the carriers, a process usually termed as excitation-induced dephasing. Secondly, phase space filling of electron–hole states (also known as Pauli blocking) as well as screening of Coulomb interactions results in reduced exciton binding energy and oscillator strength. This process manifests itself in a decrease of exciton peak area, and this causes the bleaching of resonance. Thirdly, the quasiparticle band gap energy decreases due to reduction of repulsive Coulomb interactions. Combined with decrease exciton binding energy from phase space filling and screening, there can be a shift of exciton resonance either to higher or lower energy.

In Fig. 8(b), the measured absolute magnitude of excitation-induced broadening of 6.3 × 10^−12^ meV cm^−2^ compares well to the results for monolayer MoS_2_ studied under similar experimental conditions^121^ and also for exciton–exciton scattering in WSe_2_ monolayers.^125^ The large magnitude of carrier-induced broadening, compared to typical III–V and II–VI quantum well systems, reflects the unusually strong Coulomb interaction in TMDC monolayers. It is usually difficult to differentiate the contributions of phase space filling and Coulomb screening to the bleaching signal. But recent reports^44,130,137^ show a strong modification of the optical response from the presence of free electrons in spin-split lower conduction band in WS_2_,^130^ indicating the existence of significant contributions from screening in TMDC monolayers. An instantaneous blue shift of exciton resonance, just after excitation has also been observed, subsequently turning into a red shift. The shift of the exciton resonance due to presence of carriers involves a subtle interplay between reduction of exciton binding energy and renormalization of the quasiparticle band-gap. Although the latter contributes to a red shift of the optical transitions, for optically excited^94^ and electrostatically gated samples,^10,76,130^ a blue shift of the exciton peak under non-equilibrium conditions in monolayer TMDC was previously reported. For the case of MoS_2_ monolayers,^44^ many body calculations predict an effective red shift in the presence of free electrons and holes for all but the lowest densities of monolayers. Typically some specific conditions such as, ratios of bound and unbound electron–hole pairs, carrier temperature, *etc.* influence the microscopic origin of the energy shift due to the presence of excited carriers.

The time scales of initial decays corresponding to each contribution (linewidth, area and energy) are typically of the order of 10–15 ps. Thus the probability of radiative recombination of thermalized excitons and free electron–hole (at room temperature) can be excluded due to the extreme short, picosecond time scales (theoretical predictions for effective radiative recombination are in nanosecond time scale^140,141^). Also, the scattering of bright excitons to optically dark states due to the conduction band splitting in monolayer TMDCs^51^ is expected to occur on time scales of a few picoseconds.^58^ Although this process is typically accompanied by a decrease in photoluminescence from the sample,^58,142^ it should not lead to strong modification of reflectance contrast. Non-radiative recombination *via* defects and Auger-type exciton–exciton annihilation are therefore considered to be the dominant decay channels. The decay times from curves in Fig. (8) decreased with increasing pump fluences. It can be said that overall, the presence of photoexcited carriers and their subsequent non-radiative decay (by Auger process) are responsible for initial transient changes of optical response at short times scales, <10 ps.

The regime of slower dynamics involves studying the optical response at longer delay times, typically longer than ∼100 ps, where a finite carrier population is indeed still present in the systems. Changes in optical response at later time-scales can be explained largely by phonon dynamics, that is, by heating and subsequent cooling of the lattice. Since non-radiative phenomenon contributes to the main recombination channel in currently available TMDC monolayers, it is reasonable to conclude that energy of pump pulse is largely transferred from carrier to phonon system. As for an Auger-type annihilation mechanism in which an electron–hole pair recombines by exciting a second electron–hole pair in a higher energy state, the excess energy of the second pair is rapidly converted to phonons during its relaxation towards band edge. In such cases, the energy transfer rate to the phonon system roughly matches with effective annihilation rate so that recombination of optically injected carriers correlates with increase of phonon population. Within few picoseconds, the energy is expected to be subsequently transferred from optical to acoustic phonons^143,144^ followed by the subsequent thermalization of phonons. Most importantly, the resulting heating of the lattice modifies the optical response of the material by shifting the exciton peak position to lower energies and by introducing an additional line width broadening mechanism from increased carrier-phonon scattering rates. In general, both temperature dependent shift and linewidth of the resonance can exhibit nontrivial temperature dependence. Usually for mono- and few-layered TMDs, the scaling of shift and broadening assumes a linear behavior for not too large temperatures.

An estimate of temperature increase Δ*T* can be made by assuming the total energy of the pump pulse to be converted into heat and use the relation17
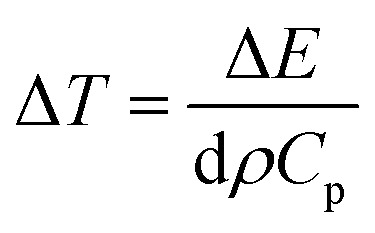
where *d* ∼ 0.6 nm denotes thickness, and *ρ* ∼ 7.5 g cm^−3^ the density of a typical WS_2_ monolayer, *C*_p_ ∼ 0.25 J g^−1^ K^−1^ is the heat capacity of bulk WS_2_ (ref. 145) and Δ*E* is the absorbed pulse energy per area (for example, using 5% absorption typically observed in such monolayers).

The results of this estimate support the fact that exciton line broadening and shift at longer time scales are due to increase in lattice temperature. In addition, subsequent cooling of the lattice (with typical time constants on the order of 100 ps) has already been reported in atomically thin materials.^146^ This rapid cooling, attributable to efficient transfer of heat from sample to underlying substrate, has been modeled by assuming that the substrate acts as cold reservoir and heat flow is limited by interfacial thermal conductance *G*.^147,148^ For the case of WS_2_ monolayer, this cooling time is given by18
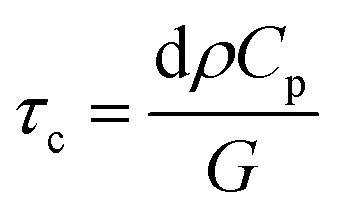
*G* being the interfacial thermal conductance varying from 0.1 to several 10 s of MW m^−2^ K^−1^,^143,146,149,150^ yielding *τ*_c_ in the range of 10 s of picoseconds to several nanoseconds. Thus at longer time scales after the excitation (>10 ps), pump induced changes in the optical response can be attributed to temperature increase of the lattice with subsequent cooling. The overall scenario after pulsed optical excitation of WS_2_ monolayer can be explained as follows: initially, after creation of electron–hole pairs by the pump pulse, broadening and bleaching of exciton resonance may occur accompanied by a small blue shift in the energy of resonance. After that, non-radiative decay channels dominated by Auger exciton–exciton annihilation contribute to decay in carrier population. This decay occurs on a fast time scale, on the order of 5–15 ps. During this process, the energy is transferred from carrier to phonon system, resulting in a local temperature increase of the lattice, thus leading to additional red shift of exciton transition. The remaining response that decays on a time scale on the order of 100 ps, reflects the cooling of the lattice by heat transfer from the monolayer to underlying substrate.

## 6. Exciton transport and related optoelectronic devices

### 6.1 Physical mechanism of electrical transport

When considering model p–n junctions from 2D materials as well as from conventional semiconductor bulk heterojunctions, the underlying physical mechanism of rectification can be different, although the shape and features of *I*–*V* characteristics between the two may be similar.

**Fig. 9:**
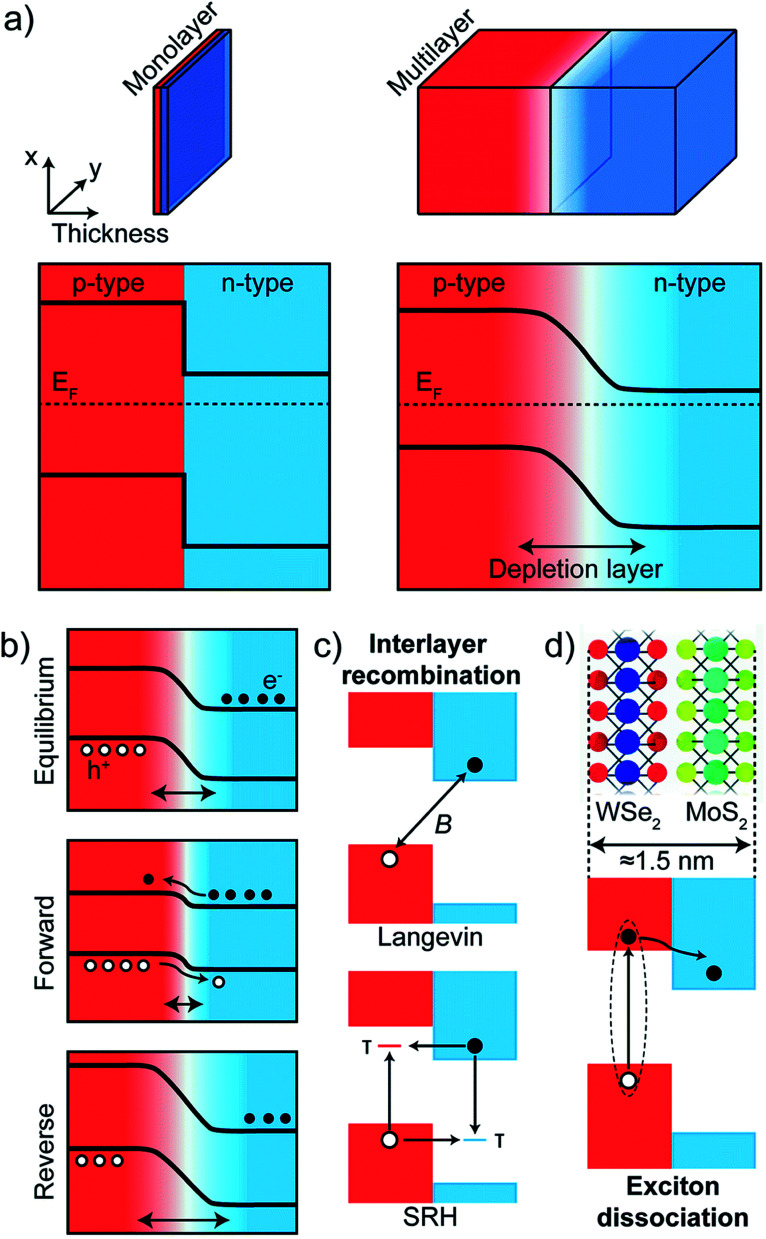
Band profiles of p–n junctions in bulk and atomically thin materials. (a) In atomically thin monolayer, there is a sharp discontinuity at the interface of a p- and n-type material (left panel) whereas band bending occurs inside the depletion region in p–n junctions of bulk conventional semiconductors. (b) Band profiles under equilibrium (no external bias), forward and reverse bias in bulk p–n junctions. (c and d) Schematic diagrams of interlayer recombination (c) in a monolayer–monolayer p–n junction and of (d) exciton dissociation process. Adapted with permission from ref. 157 Copyright 2018 Royal Society of Chemistry.

In ultrathin 2D materials, reducing the thickness of p- and n-type materials modifies the interface between two materials (Fig. 9(a)). In bulk p–n junctions, a depletion region is usually formed that is devoid of free charge carriers due to charge transfer at the interface between two materials. For atomically thin junctions, (for example, vertical junctions between monolayers of MoS_2_ and WSe_2_) such a depletion layer cannot be formed because of reduced thickness. From the band profiles (Fig. 9(a)), a sharp discontinuity of the bands of p- and n-type materials results at the interface between two monolayers (left panel), whereas in the bulk case, the bands bend in the depletion region (right panel). An application of an external bias voltage modifies the depletion region in a bulk p–n junction (Fig. 9(b)). In a forward bias, the size of the depletion region reduces, whereas it increases in a reverse bias. As forward voltage increases, the depletion region becomes thinner until the built-in electric field cannot counteract charge carrier motion across the junction. This results in an increase in current across the junction. In atomically thin junctions, the forward biased current is governed by tunnel-mediated interlayer recombination between majority carriers at the bottom (top) of the conduction (valence) band of the n-type (p-type) material. The mechanism of this interlayer recombination can be of two types: Langevin recombination, in which there is a direct recombination of an electron and hole being mediated by Coulomb interaction, and Shockley–Read–Hall (SRH) recombination, which is mediated by inelastic tunneling of majority carriers into trap states in the gap^151–153^ (Fig. 9(c)). The rectifying *I*–*V* characteristics can then be explained by an increase of interlayer recombination rate under forward bias, where the two processes can be present both at the same time in a 2D p–n junction. The photocurrent generated in a 2D junction and its dependence on a gate field can be modeled by the two recombination processes.^37^

### 6.2 Electrically driven excitonic light emission

**Fig. 10:**
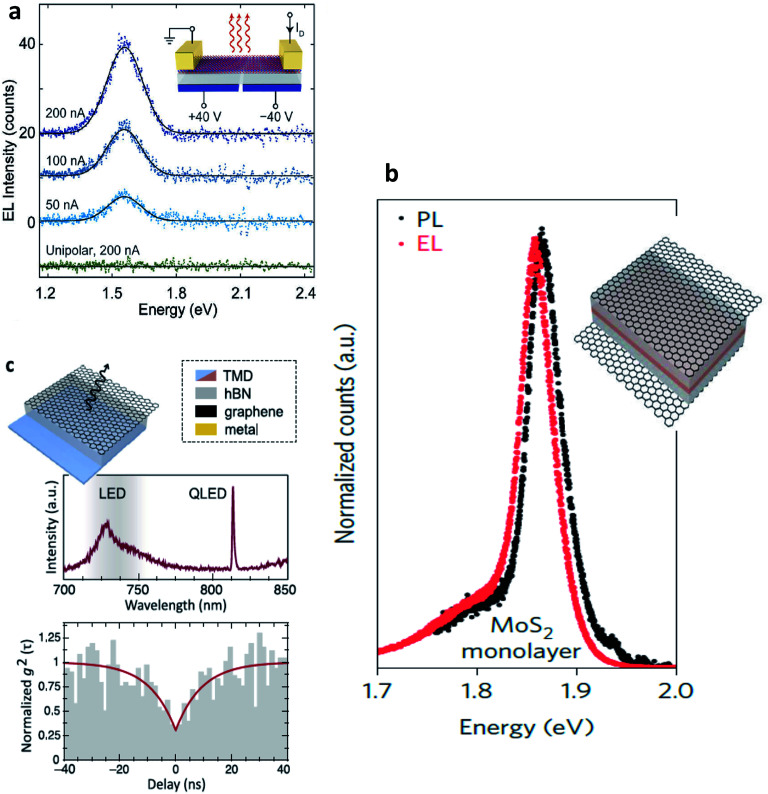
Electroluminescent devices (a) electroluminescence (EL) emission spectra of monolayer WSe_2_ electrostatic p–n junctions with split gate electrodes recorded for constant currents of 50, 100 and 200 nA. Green curve demonstrates no light emission obtained under unipolar (n-type) conduction. (Adapted with permission from ref. 34 Copyright 2014 Nature Publishing Group). (b) Comparison between EL and PL spectra of graphene/hBN/TMD/graphene vdW heterostructure. Inset shows the device structure. The light brown TMD layer is sandwiched by two hBN layers. The outermost electrodes are composed of graphene. (Adapted with permission from ref. 161 Copyright 2015 Nature Publishing Group). (c) Graphene/hBN/TMD device showing a narrow emission line from a localized exciton state (top). Photon correlation measurements of the EL showing photon antibunching (bottom). Inset shows the corresponding device structure. (Adapted with permission from ref. 178 Copyright 2016 ACS Publications).

Electroluminescence (EL) emission is a phenomenon under electrically driven excitonic light emission due to the radiative recombination of electrons and holes in the semiconductor. In 2D semiconductors, the emission can either be induced by one type of carrier (thermal population of exciton states due to Joule heating^154^ or exciton formation by impact excitation in a strong electric field^155^ or by the simultaneous injection of both carriers (electrons and holes) into the 2D material in a lateral device configuration or in a vdW heterostructure. Electrostatically defined p–n junctions with split-gate electrodes were first realized to observe this phenomenon^34,35,156,157^ (Fig. 10(a)). In a MoSe_2_/WSe_2_ heterobilayer, interlayer exciton EL was observed^158^ using the above device structure. Electron–hole pairs can also be injected in ionic liquid gated field-effect transistors (FET)^159^ or by applying an ac voltage between the gate and semiconductor.^160^ With graphene electrodes, hBN tunnel barriers and TMD monolayer semiconductor, vertical vdW heterostructure light emitters have also been demonstrated^161^ (Fig. 10(b)). The role of hBN tunnel barriers has been to prevent direct carrier tunneling. In another related device architecture,^162,163^ holes (here the minority carriers) are injected from a graphene electrode through a single hBN tunnel barrier into an electrostatically doped (n-type) TMD sheet (Fig. 10(c)). As confirmed by photon correlation measurements, this device structure allowed for quantum light emission driven by electric field, from localized exciton states.^162^ Mixed dimensional silicon/TMD vdW heterojunctions,^164,165^ in which p-doped silicon serves as a hole injection layer into n-type MoS_2_ have also been realized as exciton related EL devices. The EL quantum yield at room temperature being typically ∼1% for both lateral and vertical device geometries,^33,161,163^ is currently being limited by non-radiative recombination and is believed to increase with increase in quality of samples. Light emission at current levels as low as ∼100 pA has already been demonstrated and the emission tunable between neutral excitons and trions.^35,161^ In TMD multilayers also, EL has been observed by carrier redistribution from the indirect to direct valleys in presence of a high electric field,^166^ indirect valley filling^167^ or by the injection of hot carriers from metal TMD junction.^168^

### 6.3 Valley optoelectronic devices

**Fig. 11:**
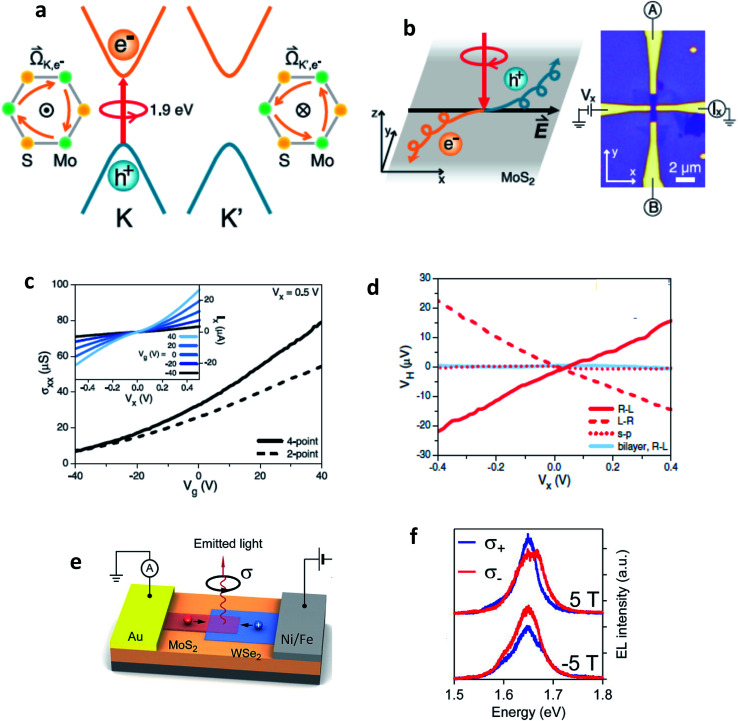
Valley Hall effect based optoelectronics. (a) Schematics of valley-dependent optical selection rules and the electrons at the K and K′ valleys that possess opposite Berry curvatures *Ω⃗*. The orange arrows represent clockwise hopping motions of the K and K′ electrons. (b) Experimental demonstration of photoinduced valley Hall effect with the image of a Hall bar device. (c) Two-point (dashed line, *V*_x_ = 0.5 V) and four-point (solid line) conductivities of the device as a function of back gate voltage *V*_g_. (Inset) Source–drain bias (*V*_x_) dependence of the current along the longitudinal channel (*I*_x_) at different back gate voltages *V*_g_. (d) In monolayer MoS_2_, the sign of photovoltage produced in a Hall bar depends on the helicity of light, whereas the effect is absent in a bilayer or for linear polarization (Fig. 10(a)–(d): adapted with permission from ref. 193 Copyright 2014 American Association for the Advancement of Science). (e) Valley polarization by spin injection. Monolayer WSe_2_ is contacted using a ferromagnetic electrode (permalloy). MoS_2_ is transferred on top of the MoS_2_ channel, forming a heterojunction diode. Under the application of a positive bias voltage to the permalloy electrode, holes are injected from the permalloy electrode and recombine in the junction with electrons injected from the MoS_2_ side, resulting in light emission. (f) Spin-polarized charge carrier injection from a ferromagnetic electrode into a vdW heterostructure light emitter leads to circularly polarized light emission that can be tuned by an external magnetic field. (Fig. 10(e) and (f): adapted with permission from ref. 178 Copyright 2016 ACS Publications).

TMD semiconductors offer the possibility of valley-selective excitation of excitons, thus utilizing the valley degree of freedom as a new paradigm for data processing. Although valley-dependent electronics and optoelectronics have theoretically been proposed in semi-metallic graphene,^63,169–171^ presence of inversion symmetry in the crystal structure of pristine graphene makes it extremely difficult for optical and electrical control of valley degrees of freedom. TMDC monolayers, for example MoS_2_, possess staggered honeycomb lattice structure, and are inversion asymmetric. Their fundamental direct energy gaps are located in the K and K′ valleys of the Brillouin zone (Fig. 11(a)). The charge carriers in K and K′ valleys experience effective magnetic fields (proportional to Berry curvature *Ω⃗*)^171^ of opposite signs due to breaking of inversion symmetry. Such a magnetic field defines optical selection rules for optical pumping of valley polarized carriers by circularly polarized photons.^16–18,172^ This magnetic field is also responsible for generating an anomalous velocity for charge carriers.^55,173^ When the semiconductor channel is biased, electrons from different valleys experience opposite Lorentz-like forces and start moving in opposite directions perpendicular to the drift current – a phenomenon known as valley Hall effect (VHE).^63,171,173,174^ Under time-reversal symmetry, equal amounts of Hall current from each valley flow in opposite directions so that no net Hall voltage is produced. The time-reversal symmetry can be broken by shining circularly polarized light onto a Hall bar device (Fig. 11(b)) and creating a population imbalance between the two valleys (valley polarization). Under a finite electrical bias, this should give rise to a net transverse Hall voltage (associated with VHE), given by^171^19
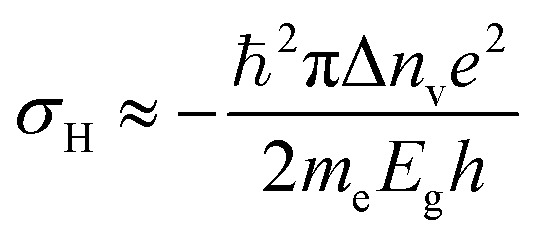
where *σ*_H_ is the anomalous Hall conductivity being linearly proportional to carrier density imbalance (Δ*n*_v_) in K(*n*_K_) and K′(*n*_K′_) valleys, *E*_g_ being the direct band gap and *m*_e_ is the effective electron mass in the conduction band of TMD material.

Generation of spin-valley coupled circular photovoltaic currents^175^ has been demonstrated in WSe_2_ and valley dependent photodetection has been observed in CVD grown MoS_2_.^176^ Most of these studies were concentrated on electric fields that dissociate excitons and drive away electrons and holes to opposite directions, giving rise to the valley Hall effect. There is also an exciton Hall effect^177^ in which statistical forces arising due to thermal/chemical potential gradients drive electrons and holes in the same direction after locally exciting a high density of valley-polarized excitons in TMD monolayer. The transport of excitons in this case, both longitudinal and transverse, can be investigated by observing the thermally driven exciton diffusion using polarization-resolved PL imaging.

There are also several reports of electrically induced valley polarization in 2D semiconductors. Being first accomplished in a lateral WSe_2_ p–n junction^167^ defined by ionic liquid gating, the EL from this device was found to be circularly polarized with the helicity depending on the current flow direction. This is attributed to different electron–hole overlaps in K and K′ valleys which can be controlled by an in-plane electric field. Owing to spin-valley locking in TMD semiconductors, there is a report of another work^178^ employing spin-polarized charge carrier injection from ferromagnetic electrode into vdW heterostructure, resulting in valley polarization. The resulting valley polarization led to circularly polarized light emission that can be tuned by external magnetic field (Fig. 10(e) and (f)).

### 6.4 Photovoltaic solar cells

TMDs provide an opportunity for the realization of ultrathin and ultralight photovoltaic devices owing to their strong absorption in the solar spectral region,^86^ favorable band gaps and high internal radiative efficiencies.^102^ Power conversion efficiencies (PCE) exceeding 25% have been predicted^179^ with TMD materials, indicating that these materials may become competitive or even outperform conventional photovoltaic technologies. Photovoltaic effects have been observed in heterostructures composed of MoS_2_ and WSe_2_ forming a type-II band alignment,^36–38^ in which lowest energy conduction band states are in MoS_2_ and highest energy valence band states in WSe_2_. The charge transfer across the junction then takes place after relaxation of photogenerated carriers driven by band offsets yielding a sizeable photovoltaic effect.^157,180–182^ Lateral p–n junctions realized by split-gate electrodes^33,34,183^ and lateral metal/TMD Schottky junctions.^184^ Carrier extraction may either occur laterally or vertically using transparent graphene electrodes. Device architectures have been explored in which chemical doping has been applied to form 2D homojunction in the same material. Examples include plasma-induced p-doping of top layers in n-type MoS_2_ multilayered crystal^185^ and mechanical stacking of n-type MoS_2_:Fe and p-type MoS_2_:Nb.^186^

TMD monolayers, despite having large optical absorption coefficients, absorb only a fraction of the incident light. Although the absorption can be increased by using multilayers, it is then limited to ∼40% by surface reflection. To overcome this limitation, light-trapping techniques, *e.g.*, by placement of a TMD semiconductor on top of metallic back reflector^187^ resulted in ∼90% broadband absorption in only 15 nm thick layer. Photogenerated excitons dissociate efficiently into free carriers by tunnel ionization in lateral device structures,^188^ or driven by band offsets that exceed the exciton binding energy of type-II vertical junctions.^189^ Large binding energies of excitons in TMD semiconductors, poor carrier transport properties,^190^ inefficient carrier extraction at the contacts leading to pile up of photocarriers in the device and recombination losses are the factors responsible for low PCEs in TMD-based photovoltaic devices.

## 7. Conclusions

Although handful of studies on exciton physics in two dimensional semiconductors have been made, versatile excitonic landscape including dark, bright, localized and interlayer excitonic states is yet to be explained. As far as optical response and non-equilibrium dynamics in these materials are concerned, the relative spectral position of dark and bright states is still unclear. Although the exciton–phonon scattering has been well understood, studies related to the impact of exciton–exciton scattering needs further investigation. Activating spin- and momentum-forbidden dark excitons in the area of exciton based device applications needs to be strategized. On the application side, wafer-scale synthesis of uniform TMD layers has been achieved,^191,192^ but the monolithic growth of large area van der Waal heterostructures continues to be challenging. Another challenging area is the lack of controllable and reliable doping scheme, which impacts detrimentally the performance of devices. For example, it is extremely difficult to create low resistance ohmic contacts due to the presence of large Schottky barriers, posing a real challenge to the process of carrier injection in TMDs. In addition, non-radiative recombination, mediated by the high defect concentration in TMDs or by biexcitonic recombination, limits the performance of optoelectronic devices. Although the former challenge can be averted by using high quality material or chemical treatment, the latter restricts the range of useful carrier concentrations.

## Conflicts of interest

There are no conflicts to declare.

## Supplementary Material
